# How to differentiate primary mucinous ovarian tumors from ovarian metastases originating from primary appendiceal mucinous neoplasms: a review

**DOI:** 10.3389/pore.2025.1612066

**Published:** 2025-05-12

**Authors:** Weronika Kawecka, Iwona Pasnik, Aneta Adamiak-Godlewska, Marek Semczuk, Magdalena Tyczynska, Andrzej Semczuk

**Affiliations:** ^1^ IInd Department of Gynecological Surgery and Gynecological Oncology, Lublin Medical University, Lublin, Poland; ^2^ Student’s Research Group at the IInd Department of Gynecological Surgery and Gynecological Oncology, Lublin Medical University, Lublin, Poland; ^3^ Department of Clinical Pathomorphology, Lublin Medical University, Lublin, Poland

**Keywords:** primary mucinous ovarian cancer, LAMN, pseudomyxoma peritonei, serum markers, tretment protocols

## Abstract

The accurate distinction between primary and secondary mucinous ovarian cancers is a crucial tool for effective surgical and systematic treatment. Mucinous ovarian metastases of appendiceal origin are a special group of tumors because they appear even in half of female patients with primary appendiceal mucinous carcinomas and demonstrate pathological similarity to primary ovarian mucinous neoplasms. The current literature review focuses on the differences based on pre-operative symptoms, radiological findings, the spectrum of microscopic features, and the significance of the immunophenotype of each tumor. Treatment options, including surgical management and adjuvant chemotherapy protocols, are also briefly overviewed. In conclusion, the source of the ovarian tumor mass might be suggested by preoperative symptoms, values of antigens, and imaging findings. However, the confirmation of the tumor origin is only made after the postoperative pathological examination. Investigating the most accurate immunohistochemical markers and new molecular features may improve diagnostic efficiency in future research.

## Introduction

Metastases to the ovaries generally originate from gastrointestinal tract tumors and often mimic primary ovarian lesions [1–3]. The special type of tumors are appendiceal mucinous neoplasms (AMNs), which are found to develop ovarian metastases in approximately 50% of cases [4, 5]. The distinction between AMNs and primary mucinous ovarian tumors is difficult and problematic because of non-specific preoperative symptoms, histological similarity, and the overlapping of immunohistochemical markers [1, 3, 6–10]. The diagnosis determines the extent of surgical treatment, the type of adjuvant chemotherapy, and the patient’s outcome.

Recently, we published a rare case study of a 61-year-old woman affected by ovarian metastatic low-grade appendiceal mucinous neoplasm mimicking the primary ovarian mucinous carcinoma [11]. We recommended that “…clinical specialists of gynecological oncology should remain conscious of the possibility of ovarian tumors of gastrointestinal origin in addition to mucinous ovarian tumors” [11]. To continue our scientific interest, we reviewed the main differences between AMNs and mucinous ovarian tumors, the possibilities of their preoperative management, difficulties with pathological confirmation of the tumor type, the treatment protocols applied worldwide, and finally, the outcome of patients.

## Classification and epidemiology

Generally, primary mucinous ovarian tumors constitute 12%–15% of all ovarian malignancies and range a spectrum of histologically different tumors, benign cystadenomas/cystadenofibromas, borderline ovarian tumors, and mucinous ovarian tumors [12–15]. While mucinous ovarian tumors are found to arise from benign and borderline precursors to high-grade neoplasms, they are rare entities (3%–10% of all primary epithelial ovarian cancer cases). They are subdivided into the expansile and infiltrative subtypes. Infiltrative histology is found in approximately 50%–60% of all reported mucinous ovarian tumors. However, it is essential to note that these findings are constrained by a limited cohort group, with lower than 50 patients included in the analysis [16]. Although younger patients (below 33 years of age) are more likely to develop benign and borderline tumor types, malignant lesions are more specific for the elderly (i.e., population above 50 years of age). However, the data presented are derived from studies that encountered specific restrictions, including a limited number of women diagnosed with malignant mucinous ovarian tumors, participants from a single Institution, and a short follow-up [1, 12–14, 17].

Metastatic ovarian tumors account for 5%–30% of all malignant ovarian neoplasms [7, 18–21]. Secondary mucinous ovarian tumors primarily originate from the breast, the colon, and the stomach, while the appendix is the origin of metastases in 3%–7% of cases [7, 18]. The limitation of this information is that it encompasses patients from a specific geographic region (the Netherlands), and a defined time frame (2000–2010) [18]. Generally, patients with tumors originating from the gastrointestinal tract tend to be older than those whose tumors originate from outside this tract [21]. Additionally, patients with primary epithelial ovarian cancer are generally older than those who were diagnosed with ovarian metastases [21, 22].

Appendiceal mucinous neoplasms involve simple mucoceles, serrated polyps, low-grade appendiceal mucinous neoplasms (LAMNs), high-grade appendiceal mucinous neoplasms (HAMNs), and adenocarcinomas (Table 1). The reliable epidemiological data are not well-proven because the applicable definitions are inconsistent. While appendiceal mucinous neoplasms account for less than 1% of all cancer cases, appendiceal tumors are diagnosed in approximately 0.4%–1.7% of patients after appendectomies [8, 25–28]. The peak incidence of AMNs occurs in the sixth decade of human life [8]. Female patients are more likely to develop the disease (50%–55% of the appendiceal tumor population) [26]. Approximately 50% of female patients with AMNs develop metastases to the ovaries [4, 5].

**TABLE 1:** Pathological features of appendiceal mucinous neoplasms (AMNs) serrated polyps, low-grade appendiceal mucinous neoplasm (LAMN), high-grade appendiceal mucinous neoplasm (HAMN), adenocarcinoma), pseudomyxoma peritonei (PMP) and primary mucinous ovarian tumors (benign tumor, borderline tumor, adenocarcinoma) [8, 12, 14, 20, 23, 24].

Tumor type	Characteristic features
Serrated polyps	• Lacking cytological dysplasia • Serrated lesions: dysplastic polyps (serrated adenoma-like dysplasia, serrated-type dysplasia, adenoma-like dysplasia) and non-dysplastic polyps
LAMN	• Non-infiltrative invasive mucinous neoplasms with low-grade cytological atypia and any of the following characteristics: loss of the muscularis mucosae and lamina propria, fibrosis of submucosa, different forms of “pushing” invasions (expansile or diverticulum-like growth), dissection of acellular mucin in the wall, different patterns of epithelial growth (undulating or flattened epithelial growth), rupture of the appendix, and mucin, and/or cells outside the appendix • Small, uniform, darkly stained, basally orientated nuclei which showed nuclear polarity with large cytoplasmic mucin in neoplastic cells
HAMN	• Features histologically similar to LAMN • Neoplastic epithelium with unequivocal high-grade features (vesicular enlarged nuclei with full-thickness stratification, loss of nuclear polarity, numerous mitotic figures, and prominent nucleoli)
Appendiceal adenocarcinoma	• Infiltrative invasion (desmoplastic reaction, small angulated irregular glands or tumor budding) • Well-differentiated adenocarcinoma: neoplastic epithelium with minor nuclear atypia lining the cystic mucin pools • Poorly differentiated: no or little gland formation • Adenocarcinoma with signet ring cells: <50% signet ring cells present • Signet ring cell carcinoma: >50% signet ring cells present
PMP	• Acellular mucin: mucin without neoplastic epithelial cells • Low-grade mucinous carcinoma peritonei: minor cytologic atypia, sporadic mitosis, “pushing” invasion, strips, gland-like structures or small clusters of cells • High-grade mucinous carcinoma peritonei: more cellular, cribriform growth, high-grade cytological atypia, more mitoses, destructive infiltrative invasion • High-grade mucinous carcinoma peritonei with signet ring cells: any lesion with a component of signer ring cells
Benign mucinous ovarian tumor (cystadenoma, cystadenofibroma)	• Thin-walled cysts lined by a single layer of mucinous columnar cells with basally oriented nuclei • Fibrous stroma • Possibility of small papillary formations • Rare (<10%) epithelial proliferation, stratification, and branching papillae
Borderline tumors	• Multicystic tumor containing intracystic papillae without architectural complexity (at least 10%) • Stratified proliferative epithelium with low-to-moderate atypia • Possibility of stromal microinvasion (<5 mm) made of single cells, cell nests, confluent glandular structures, or a cribriform growth pattern
Ovarian adenocarcinoma	• Expansile: absence of destructive stromal invasion or stromal reaction, with confluent or complex malignant glands, with or without minimal intervening stroma, the possibility of the focal area of infiltrative-type invasion (<5 mm) • Infiltrative: presence of glands, cell clusters, or individual cells; disorderly infiltrating the stroma; desmoplastic stromal reaction >5 mm

Pseudomyxoma peritonei (PMP) is an uncommon condition, that is not assigned to AMNs but contains mucinous ascites, spreads to peritoneum, and originates from a perforated AMN (particularly, LAMN). This condition rarely develops from primary mucinous ovarian neoplasia, and when it does, the lesion is a mature teratoma [25]. The prevalence of pseudomyxoma peritonei is truly difficult to determine, but it is said to affect 22 people per million per year [29]. Interestingly, the presence of PMP is correlated with the severity of AMN. Disseminated peritoneal mucinous spread predicts the advanced stage of the disease and metastatic occurrence [6].

## Clinical symptoms

The clinical symptoms of primary ovarian tumors and AMNs are non-specific and appear in 70% of patients. They include abdominal pain, postmenopausal vaginal bleeding, ascites, abdominal distention, anemia, and rapid weight loss [28, 29]. Postmenopausal vaginal bleeding or changes in menstrual habits are also correlated with ovarian cancer. Ascites is more common for metastases than for primary mucinous ovarian tumors, and it is correlated with the advanced stage of the disease. At the same time, AMNs often present an acute-appendicitis-like pain in early stages of the disease [21, 30]. The large tumor size alone during the physical examination suggests the primary mucinous histologic subtype, while metastases to the ovary are more likely to be relatively smaller and bilateral [31]. Approximately 30% of patients with AMNs receive a preoperative diagnosis of acute appendicitis. Unfortunately, none of the symptoms presented above sufficiently represent any of these tumors [21, 30–35].

## Ultrasonography (USG) and radiological findings

In women with adnexal mass symptoms, the imaging modality of choice is to perform the pelvic ultrasonography, typically transabdominal or transvaginal, with color/power Doppler imaging [31, 36]. It is useful for determining the anatomical origin of adnexal masses, as well as for diagnosing simple cysts, hemorrhagic cysts, ovarian endometriomas, and mature teratomas [31, 37]. The USG examination is sensitive for detecting malignant lesions and might be applied to distinguish them from benign diseases with color Doppler imaging [31, 38–40]. The ovarian mucinous elements in the USG are demonstrated as “low-level” echoes and rarely contain calcifications or papillary projections within the lumen cyst [31].

The next step in the differential diagnostic process is to conduct MRI or CT scans. These imaging techniques can be useful for fully assessing large adnexal masses, identifying the primary site of their origin, and differentiating between malignant and benign lesions [39, 40]. A thick irregular wall, thick septa, papillary projections, and large soft-tissue components with necrosis strongly suggest a malignant process. In addition, the advanced stages of the disease are characterized by ascites, lymphadenopathy, ancillary findings suggestive of pelvic organ invasion(s), and the disease spread to the peritoneum and/or omentum [31, 41].

The imaging of AMNs consists of identifying neoplastic mucoceles in the USG, CT, or MRI scans. An extra-appendiceal mucin in the peritoneal cavity outside the right lower quadrant indicates the presence of PMP. This may exhibit a more varied appearance with loculated areas, displaying septations and/or curvilinear or amorphous calcifications within the mucinous implants [42].

## Serum markers

Assessing the levels of serum cancer antigen 19-9 (CA19-9), cancer antigen 125 (CA125), and carcinoembryonic antigen (CEA) is a “gold” standard in preoperative procedures. The elevation of these antigens, combined with the imaging findings, may suggest the possible origin of mucinous ovarian tumors and their malignant potential. The best predictor of a borderline or malignant tumor is an elevated level of CA125 (>35 U/mL) [43, 44]. At the same time, some data suggests that CA19-9 may also predict ovarian malignancy, especially when the CA125 level is within the normal range [45]. The CEA level is more likely to be elevated in mucinous ovarian tumors (88% of cases) than in non-mucinous (19% of cases) ovarian tumors [20]. In patients affected by mucinous ovarian tumors, the higher level of CEA, compared to CA125 and CA19-9, was observed thereafter. CEA alone is generally sufficient for distinguishing between primary ovarian tumors and metastases indicating gastrointestinal origins, but the combined assessment of the CA125/CEA ratio is also recommended [21, 46–48].

Recent literature data has focused on the role of human epididymis secretory protein 4 (HE4), which seems to be the best diagnostic predictor of epithelial ovarian cancer in premenopausal women [49]. Moreover, monitoring of combined HE4 and CA125 levels during chemotherapy is also recommended because their variations are prognostic markers [49, 50]. Another prognostic biomarker in epithelial ovarian cancer is the D-dimer level because its high pre-treatment level is associated with an unfavorable patient’s outcome [51]. Despite the above, these antigens are mostly used as prognostic factors, not during diagnostic differentiation [3, 21, 43, 46–48, 52].

**FIGURE 1:**
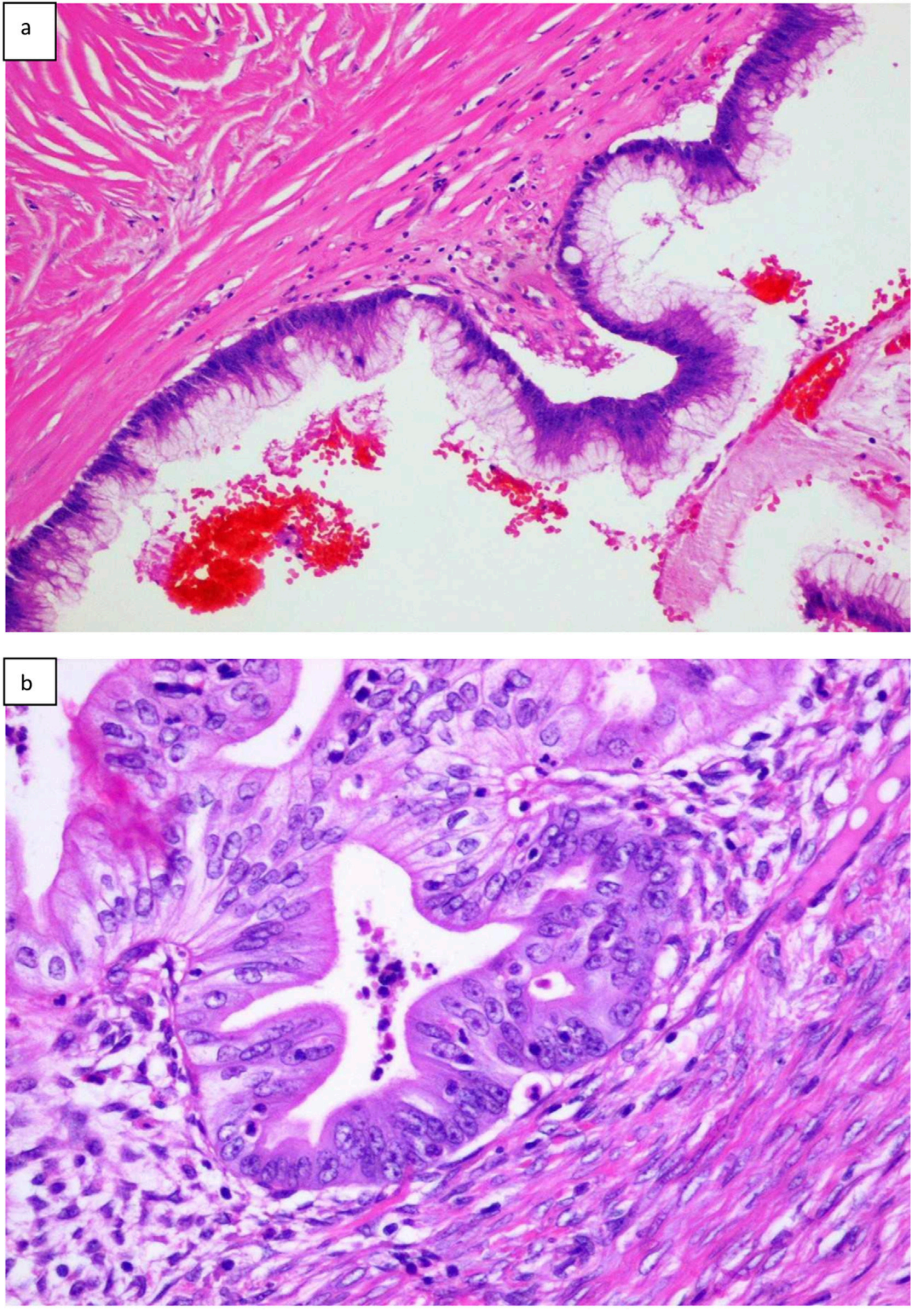
Histopathologic presentation of primary LAMN **(A)**, and primary mucinous ovarian cancer **(B)** (hematoxilin and eosin; original magnification ×100).

Interestingly, the preoperative procedures, such as the patient’s clinical profiling, imaging findings and the levels of antigens, might suggest the possible origin of mucinous ovarian tumor. Still, the postoperative pathological examination combined with immunohistochemical markers enables determining the accurate and final diagnosis [28, 53, 54] (Figure 1). Difficulties with the differential diagnosis for pathologists arise from the fact that AMNs represent a range of morphological features and may imitate primary ovarian lesions as well [3, 12, 23, 55]. Furthermore, MOCs can be divided into infiltrative and expansile subtypes. It is important to note that these subtypes may be misinterpreted by pathologists due to the presence of grade 3 nuclear atypia or microfoci that display an infiltrative invasion pattern. Infiltrative MOCs have significantly poor patient’ outcomes compared to the expansile subtype [14].

## Immunohistochemical assessment

The pathological examination must be completed by applying the panel of immunohistochemical markers, which is a valuable tool in distinguishing between AMNs and primary mucinous ovarian tumors. The immunohistochemical markers cannot be analyzed separately because they overlap in both types of tumors. For example, CDX2 is typically positive in gastrointestinal tumors but may be stained positive in primary mucinous ovarian tumors [23]. Moreover, although PAX8 is considered to be the most specific marker in primary mucinous ovarian tumors, it shows positivity only in approximately 10%–40% of cases [1, 23, 24]. For this reason, the most common panel includes a combined assessment of CK7, CK20, CDX2, PAX8, and SATB2 [1, 20, 56–62].

The current literature focuses on the application of SATB2, which seems to be the most specific marker for ovarian metastases originated from the gastrointestinal tract, even though the available data are limited due to the limited number of patients included in the analyses (only 7 cases) [56, 61–65]. In general, the typical primary mucinous ovarian carcinomas are CK7-positive, with diffuse co-expression of CK20 and CDX2, PAX8 and SATB2-negative, while AMNs are CDX2, CK20, and SATB2-positive, and PAX8 and CK7-negative. In addition, PAX8 immunopositivity strongly suggests the malignant ovarian origin of the lesion [56, 60–62]. Due to the unexpected and problematic occurrence of immunohistochemical markers in primary mucinous ovarian tumors and AMNs, the most accurate panel of markers has yet to be recommended [1, 23]. The presence or absence, and the incidence of the most common IHC markers in AMNs and primary mucinous ovarian tumors are outlined in Table 2.

**TABLE 2:** The percentage of immunohistochemical markers and their average incidence in low-grade appendiceal mucinous neoplasms (LAMNs), high-grade appendiceal mucinous neoplasms (HAMNs), primary mucinous appendiceal adenocarcinomas and mucinous ovarian carcinomas (MOCs) [10, 20, 23, 56, 61, 62, 65, 66].

	Immunohistochemical markers	Average incidence (%)
LAMN/HAMN	CK20 CDX2 CK7 SATB2 PAX8 P53	90–100 92–100 14–36 96–100 - -
Primary mucinous appendiceal adenocarcinomas	CK20 CDX2 CK7 SATB2 PAX8 P53	96–100 93 28–50 83–100 - 40
MOC	CK20 CDX2 CK7 SATB2 PAX8	33–73 33–36 97 - 10–40

The immunohistochemical markers, less common in average practice, are represented by math1, MUC1, SMAD, P53, or PGP [23]. Although they are not typically applied, some of them seem to differ between various appendiceal neoplasms. More specifically, MUC1 is found to be overexpressed (∼17%) in appendiceal adenocarcinomas, compared to LAMNs (0%), whereas SMAD4 is significantly expressed in adenocarcinomas (19%), but not in low-grade tumors. Therefore, the increased levels of those two markers may suggest the diagnosis of adenocarcinoma [23, 67, 68]. Moreover, PAX8 is more commonly expressed in expansile MOCs than in infiltrative ones, but this association is not of significant value [16].

## Molecular markers

The molecular features found in primary mucinous ovarian tumors and AMNs shed *new* light on the diagnostic path (Table 3). The typical mutations in AMNs and PMP appear in *KRAS, GNAS,* and *TP53*, but their prevalence is slightly different in the subtypes of AMNs and PMP [6, 69–71]. *KRAS* and *GNAS* are overexpressed in LAMNs at 72% and 44%, respectively, and in HAMNs and adenocarcinomas at 50% and 27% [6, 72]. Additionally, *TP53* alterations are more prevalent in HAMNs and adenocarcinomas than in LAMNs [69, 71]. Moreover, the acquisition of *TP53* mutations by HAMN may drive its progression to a more advanced clinical stage and thus might show aberrant protein immunostaining as well [73]. Moreover, molecular profiling of various appendiceal lesions reported different hotspot mutational profiling in selected genes, including *RNF1*, *SMAD4*, *KRAS*, *NRAS*, *BRAF*, and *PIK3CA* [74]. Finally, 1 *KRAS* mutation, 2 *PIK3CA* mutations, and 1 *BRCA2, EP300, TGFBR2, CHD4, CREBBP, FANCC*, and *PKHD1* mutation were reported in a case of high-grade appendiceal mucinous neoplasm mimicking tubovillous adenoma [75].

**TABLE 3:** Molecular alterations and their incidence in low-grade appendiceal mucinous neoplasm (LAMN), high-grade appendiceal mucinous neoplasm (HAMN), appendiceal adenocarcinoma, pseudomyxoma peritonei (PMP), and mucinous ovarian tumors (MOTs) [6, 69–81].

Tumor type	Molecular alterations incidence	
	High	Low
LAMN	*KRAS* (61.1%–100%), *GNAS* (63%)	RNF43, *TP53*, *BRAF*, *APC, PIK3CA, APC, FBXW7, PTEN, SMAD4*
HAMN	*KRAS* (50%–100%), RNF43 (66.7%), *GNAS* (56%)	*TP53, BRCA2, EP300, TGFBR2, CHD4, CREBBP, FANCC, PKHD1, PIK3CA, APC, FBXW7, PTEN, SMAD4*
Appendiceal adenocarcinoma	*KRAS* (44%–70%), RNF43 (33.3%), *GNAS* (27%)	*TP53*, *PIK3CA*, *APC, PIK3CA, APC, FBXW7, PTEN, SMAD4*
PMP	*KRAS* (38%–100%), *GNAS* (17%–100%), *TP53* (5%–23%)	Data not shown
MOTs	*CDKN2A* (76%), *KRAS* (64%), *TP53* (64%)	*RNF43*, *BRAF*, *PIK3CA*, and *ARID1A* (8%–12%)

There is no significant difference in *KRAS*, *GNAS*, and *TP53* alterations between primary and metastatic AMNs. However, ovarian metastases may show 22% SMAD2 expression and 16% SMAD4 point mutations [70, 76]. In addition, primary appendiceal adenocarcinomas are usually reported with *PIK3CA*, *P53*, and *APC* gene mutations, while LAMNs are usually wild-type for *BRAF*, *APC*, and *TP53* [23]. For women with PMP, the most frequently identified somatic gene mutations are *KRAS* (38%–100%), *GNAS* (17%–100%), and *TP53* (5%–23%). The impact of these mutations on the patient’s survival rate is still unresolved and the lack of their prognostic utility is highlighted worldwide [69–71]. The spectrum of mutations in primary mucinous ovarian tumors is more variable than in AMNs and it is proven that the genetic profile is unique [20]. *KRAS* mutations and *CDKN2A* inactivation are characteristic of benign and borderline primary mucinous ovarian tumors, although the copy number alterations are higher in BMOTs [77].

The most common genetic events in MOCs include copy number losses, mutations in *CDKN2A* (76%), and alterations in *KRAS* and *TP53* (64% in each case) [77, 82]. Other less frequent mutations in MOCs include *RNF43*, *BRAF*, *PIK3CA*, and *ARID1A* (8%–12%) [77–80, 82]. Moreover, *TP53* alterations and copy number aberrations are key drivers during ovarian cancer development and progression and they are truly associated with worse prognosis in MOC patients. Finally, a subset of primary mucinous ovarian adenocarcinomas (10%–15%) displayed *HER2/neu* amplification [12, 77, 81].

## Treatment protocols

The accurate diagnosis is a clue for the appropriate treatment protocol because it differs in AMNs and primary mucinous ovarian tumors (Table 4). The preoperative results only suggest the source of tumor origin. Still, the definitive diagnosis is established by a post-operative pathological assessment. Generally, the cytoreductive surgery, which involves removing all visible tumor lesions aiming for a microscopic residual disease, is the first step and still the “gold” standard in both types of tumors [20, 21, 83]. However, the surgical treatment depends not only on the tumor stage but also on the patient’s general condition and childbearing desire. Women affected by stages IA and IB and with a desire to have offspring require unilateral salpingo-oophorectomy with comprehensive surgical staging. When a candidate with stage IA-IV is eligible for surgical intervention and optimal cytoreduction is attainable without the need for fertility preservation, a total hysterectomy with salpingo-oophorectomy should be performed, including comprehensive surgical staging and debulking surgery. A poor surgical candidate with a low likelihood of optimal cytoreduction should be referred to neoadjuvant therapy with poly-adenosine diphosphate-ribose polymerase (PARP) inhibitors [17, 84, 85]. The next step after the surgery is the application of adjuvant chemotherapy, mainly hyperthermic intraperitoneal chemotherapy (HIPEC), but this procedure requires the final pathological examination of the tumor type. To avoid a “redo” surgery, the laparoscopic exploration of the abdominal cavity is also recommended [28].

**TABLE 4:** A comparison of appendiceal mucinous neoplasm and primary mucinous ovarian tumor management based on a literature review.

The procedure	AMN	Primary mucinous ovarian tumor
CRS	Should be performed	Should be performed
Appendectomy	Should be performed	Consider appendectomy if there is a suspicion of an appendiceal growth
Lymphadenectomy	Consider in an advanced-stage disease	Consider in an advanced-stage as well as early-stage disease; should be performed in confirmed infiltrative MOC
Right-sided hemicolectomy	Consider in LAMNs with a perforated appendix or with positive margins after appendectomy; should be performed in HAMNs and adenocarcinomas	Depends on the tumor size and suspected tumor origin
Chemotherapy	HIPEC (oxaliplatin or mitomycin C)	Platinum-based chemotherapy (carboplatin with paclitaxel); consider HIPEC, XELOX BCCA, and FOLFOX BCCA protocols in an advanced-stage MOC

Abbreviations: CRS, cytoreductive surgery; HIPEC, hyperthermic intraperitoneal chemotherapy; LAMN, low-grade appendiceal mucinous neoplasm; HAMN, high-grade appendiceal mucinous neoplasm; MOCs, mucinous ovarian carcinomas [1, 13, 17, 20, 28, 42, 52, 83–86].

It is worth noting that the tumor subtype also determines the surgical procedure that should be performed. For localized LAMNs, appendectomy is generally sufficient [28, 42, 86]. Right-sided hemicolectomy is not a standard procedure in LAMNs due to the fact that the incidence of positive lymph nodes reaches only 6%. This treatment should be considered if there is a perforation of the appendix during surgical intervention or if the surgical margins are not fully resected during appendectomy [86–88]. If the presence of a minor peritoneal disease in a LAMN patient is confirmed during the preoperative examination, the “one-time” laparoscopic cytoreductive surgery/HIPEC protocol should be considered; however, the results of this study are based on a small cohort [89]. For HAMNs, a right-sided hemicolectomy is recommended because the lymph node involvement may increase to nearly 30% altogether [86]. Appendiceal adenocarcinomas require right-sided hemicolectomy and regional lymphadenectomy [28, 42]. Omentectomy should be considered during cytoreductive surgery if the peritoneal spread/PMP is suspected or gross metastases are absent [90, 91].

In mucinous ovarian tumors, especially when cancer is suspected, appendectomy and peritonectomy should always be considered. Routine appendectomy is a controversial procedure. Although some data recommend omitting this procedure if the appendix appears grossly normal, especially when the gross metastatic disease is not identified, others highlight that a metastatic disease may also be present in the normal-looking appendix. The most optimal recommendation is to routinely evaluate the appendix intra-operatively. Although the studies exhibit certain limitations, including the retrospective nature of the clinical data [20, 52, 92–96]. Finally, lymphadenectomy is not a routine procedure because of a very low (0%–2%) incidence of lymph node metastasis in MOCs. However, recent studies have demonstrated several limitations, including incomplete information regarding lymph node status in patients, a small group of patients, and the reliance on data from a single institutional cohort [97–100]. Research indicates that in the advanced stages of MOCs, the systematic pelvic and para-aortic lymphadenectomy of healthy lymph nodes does not contribute to improved overall survival or disease-free survival. Furthermore, this surgical intervention may be associated with an increased incidence of several post-operative complications [52, 101]. In addition, in infiltrative MOCs, lymph node metastases might be present in approximately 30% of cases [102]. Considering the difficulties with determining the MOC subtype intra-operatively, the decision to perform lymphadenectomy should be strictly individualized. The role of routine lymphadenectomy in an early-stage disease is not clear yet [20, 52, 84]. In addition, benign mucinous ovarian tumors should be treated by resecting pathological masses, while unilateral salpingo-oophorectomy or ovarian cystectomy, cytologic washings, omentectomy, peritoneal biopsies, and routine lymphadenectomy are not yet recommended [103].

The introduction and widespread use of intraoperative hyperthermic intraperitoneal chemotherapy have significantly impacted the treatment of AMNs and PMP. HIPEC has shown a marked improvement in prognosis, clinical outcomes, and quality of life for patients, especially those diagnosed with PMP. Evidence suggests that HIPEC may have curative potential in select cases, with success rates as high as 70%–80%. However, the number of patients included in these studies is estimated to be slightly over 100 [104–106]. Combining cytoreductive surgery with hyperthermic intraperitoneal chemotherapy and applying oxaliplatin or mitomycin C seems to be the most effective treatment for PMP and advanced-stage primary appendiceal mucinous adenocarcinomas. In addition, HIPEC should be considered in all LAMN, HAMN, and metastatic AMN [20, 28, 89]. Although platinum-based chemotherapy, particularly in combination of carboplatin and paclitaxel, is the “gold standard” for all primary mucinous ovarian tumors, MOCs are less sensitive to this treatment [20, 52, 107, 108]. The role of adjuvant chemotherapy in early-stage MOCs is still under investigation [20, 52, 84, 92]. The role of HIPEC in primary mucinous ovarian tumors has been clarified in recent years. The biological similarity of primary mucinous ovarian tumors and AMNs suggests the utility of HIPEC in advanced-stage cases, particularly during interval debulking surgeries [21, 52, 92]. However, the use of HIPEC generally remains controversial. Alternative chemotherapy protocols include FOLFOX BCCA (oxaliplatin, leucovorin, and 5-fluorouracil) and XELOX BCCA (oxaliplatin and capecitabine). The response rate reaches 30% in the FOLFOX BCCA protocol, whereas no data have been documented for XELOX BCCA yet [17, 20, 52, 92].

**FIGURE 2:**
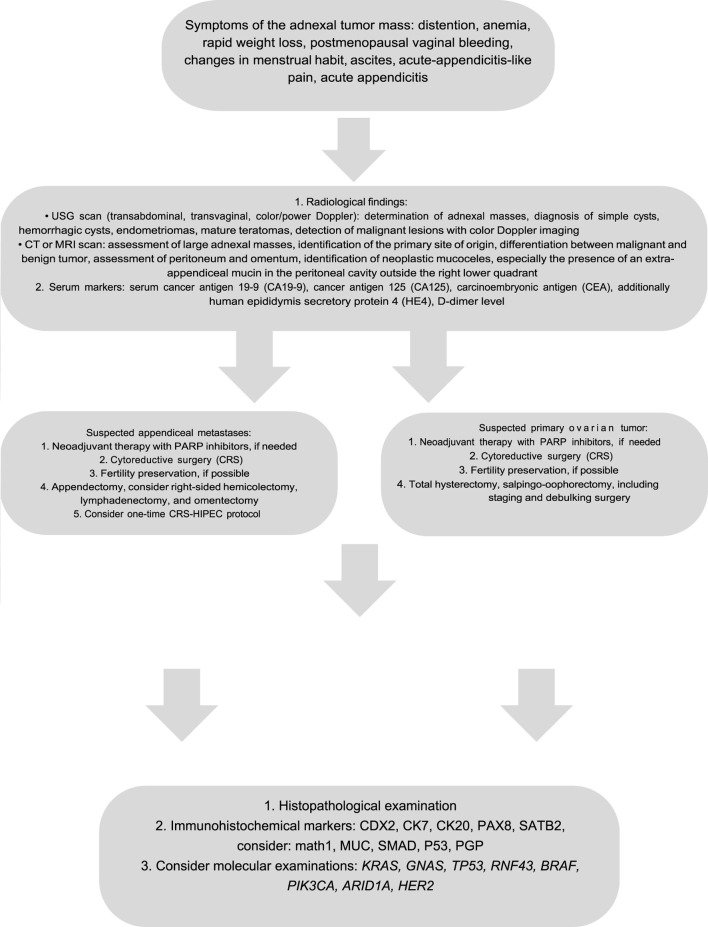
Algorithm of the diagnostic and therapeutic path in primary and secondary mucinous ovarian tumors.

Recent studies have focused on targeted therapies in early and advanced-stage primary mucinous ovarian tumors [109–111]. The efficacy of PARP inhibitors has been established in the treatment of non-mucinous epithelial ovarian tumors; however, this is inadequate for primary mucinous ovarian tumors as these tumors are not associated with *BRCA* mutations [17, 20, 52, 110]. The VEGF inhibitor (bevacizumab) and HER2 monoclonal antibody (trastuzumab) are shown to improve the overall survival rates in MOC patients. Cetuximab, the EGFR monoclonal antibody, seems to be capable of anti-proliferative activity in MOC cell-lines, which do not harbor *KRAS* mutations [17, 20, 52, 84, 92, 111–120]. Nevertheless, it is important to acknowledge that most of the studies are limited in scope and consist of small patients’ number. An algorithm briefly summarizing the diagnostic and clinico-pathological features of ovarian masses is presented at Figure 2.

## Patients’ survival

The patients’ survival depends on many clinical variables including the tumor type and clinical stage, the presence of metastases, the surgical treatment, as well as the response to chemotherapy. Generally, in the case of primary mucinous ovarian tumors, complete surgical resection is recommended in benign tumors such as cystadenoma and cystadenofibroma [12]. Benign mucinous ovarian tumors have also an excellent survival rate of >90% during a 5-year follow-up [103]. Although early-stage MOCs have an excellent prognosis (>90% in 5-year overall survival), survival in an advanced-stage disease with the existence of metastases ranges from 12 up to 30 months [12, 20]. Moreover, progression-free survival differs significantly between expansile and infiltrative MOCs. During 3-year observation, progression-free survival is approximately 90%–95% in the expansile group vs. 60%–65.5% in the infiltrative subgroup, while overall survival showed no significant differences (88.8%–96% and 87%–90%, respectively) [16, 121, 122].

The survival rate in AMNs depends on tumor progression to an advanced stage disease, which occurs in 2% of benign lesions and up to 23% of mucinous adenocarcinomas. Without the progression, the 5-year survival of patients with benign tumors reaches 100%, while for patients with malignant ones, it ranges from 30% to 80%. In PMP, overall survival ranges from 23% to 77% [44, 123–125].

Finally, the median overall survival for woman affected by ovarian metastases of colorectal origin is 17.5 months, ranging only from 3.1 months in patients without treatment to 34.1 months in women undergoing cyto-reductive surgery/HIPEC [120]. It is crucial to highlight that the studies referenced above comprised only limited number of patients, thereby limiting the scope and generalizability of their findings.

## Conclusion

The diagnosis of primary mucinous ovarian tumor is a huge challenge for gynecological oncologists, general surgeons, and pathologists. The preoperative symptoms, the antigen levels, and the imaging assessments, even when considered altogether, are not sufficient to confirm the exact tumor type. Still, they may suggest the source of the ovarian tumor mass origin. To shorten the diagnostic path and avoid repeated surgical interventions, laparoscopy might be useful procedure for localized tumors. Investigations of the most accurate immunohistochemical markers and different molecular features seem to be the most promising tools during the diagnostic differentiation. However, the necessity of research on this matter still needs to be highlighted.

## Abbreviations

AMN, Appendiceal Mucinous Neoplasm; BCCA, British Columbia Cancer Agency; BOT, Borderline Ovarian Tumor; CEA, Carcinoembryonic Antigen; CRS, Cytoreductive Surgery; CT, Computed Tomography; HAMN, High-grade Appendiceal Mucinous Neoplasm; HIPEC, Hyperthermic Intraperitoneal Chemotherapy; LAMN, Low-grade Appendiceal Mucinous Neoplasm; MOC, Mucinous Ovarian Carcinoma; MRI, Magnetic Resonance Imaging; PARP, Poly-Adenosine diphosphate-Ribose Polymerase; PMP, Pseudomyxoma Peritonei; USG Ultrasonography.
